# CRISPR-Cas, a robust gene-editing technology in the era of modern cancer immunotherapy

**DOI:** 10.1186/s12935-020-01546-8

**Published:** 2020-09-15

**Authors:** Seyed Mohammad Miri, Elham Tafsiri, William Chi Shing Cho, Amir Ghaemi

**Affiliations:** 1 Department of Chemistry, Sharif University of Technology, Tehran, Iran; 2 Molecular Medicine Department, Biotechnology Research Center, Pasteur Institute of Iran, Tehran, Iran; 3 Department of Clinical Oncology, Queen Elizabeth Hospital, Hong Kong, China; 4 Department of Virology, Pasteur Institute of Iran, Tehran, P.O.Box: 1316943551, Iran

**Keywords:** Cancer immunotherapy, CRISPR-Cas, Cas9, TCR T-cell, CAR T-cell, Allogeneic T-cell

## Abstract

Cancer immunotherapy has been emerged as a promising strategy for treatment of a broad spectrum of malignancies ranging from hematological to solid tumors. One of the principal approaches of cancer immunotherapy is transfer of natural or engineered tumor-specific T-cells into patients, a so called “adoptive cell transfer”, or ACT, process. Construction of allogeneic T-cells is dependent on the employment of a gene-editing tool to modify donor-extracted T-cells and prepare them to specifically act against tumor cells with enhanced function and durability and least side-effects. In this context, CRISPR technology can be used to produce universal T-cells, equipped with recombinant T cell receptor (TCR) or chimeric antigen receptor (CAR), through multiplex genome engineering using Cas nucleases. The robust potential of CRISPR-Cas in preparing the building blocks of ACT immunotherapy has broaden the application of such therapies and some of them have gotten FDA approvals. Here, we have collected the last investigations in the field of immuno-oncology conducted in partnership with CRISPR technology. In addition, studies that have addressed the challenges in the path of CRISPR-mediated cancer immunotherapy, as well as pre-treatment applications of CRISPR-Cas have been mentioned in detail.

## Background

According to statistics, the appearance of about 18.1 million newfangled cancer victims and 9.6 million cancer-related fatalities just in 2018 is entirely self-explanatory of the importance of developing more efficient cancer therapy strategies [1]. Besides the well-known approaches of cancer therapy such as chemotherapy, radiotherapy, surgery, as well as the latest methods such as oncolytic virotherapy, harnessing the immune system against cancer cells has been developed [2, 3]. Engineered T-cell’s anti-cancer properties have expanded the application of immunotherapy from viral infections to cancer treatment [4]. Adoptive cell transfer (ACT) cancer immunotherapy can be done through deployment of three different immunogenic constructs including tumor-infiltrating lymphocytes (TILs), T-cell receptor (TCR) T-cells, and engineered chimeric antigen receptor (CAR) T-cells [5]. To achieve desired CAR T-cells or to modify TCR T-cells, incorporation of a gene-engineering tool is needed. Clustered regularly interspaced short palindromic repeats (CRISPR) in association with Cas nuclease stands out from other gene-editing methods, such as zinc finger nucleases (ZFNs) and transcription activator-like effector nucleases (TALENs), due to simplicity, high fidelity, and multi-target editing potential [6]. The role of CRISPR is not limited to therapeutic purposes. CRISPR screening technology is applied to find novel immunotherapy targets and other unknown genetic participants in immune response pathways. The outcome of those screening trials constitute an integral part of future cancer immunotherapy approaches with improved fidelity and efficiency, as well as minimal off-targeting and side-effect problems [7].

To address the chronic malignancy of cancer, many ongoing pre-clinical and clinical trials have been applying constantly. In this article, we focused on an exclusive zone of these trials in which adoptive immunotherapy intersects with a sophisticated gene-editing tool, CRISPR-Cas technology. However, a huge tendency toward utilization of this combination has been aroused some ethical controversies [8]. Humanitarian health concerns, as well as other limitations associated with CRISPR-assisted cancer immunotherapy, and the attempts to bypass these challenges have not been overlooked from our critical viewpoint.

## Cancer immunotherapy, from genesis to modern CARs

As it is obvious from the phraseology, cancer immunotherapy stands for all cancer therapeutic procedures in co-operation with the immune system. One of the earliest reports of immune system’s triumph against cancer backs to 1890, when William Coley observed that some cancer patients with skin infection experience better condition than those without infections, a phenomenon that later determined was due to the immune responses elicited by bacterial infection [9]. Immunological-assisted cancer therapy remained a controversial subject for decades until 1965, when leukemia cell’s regression of a patient was reported following bone marrow transplantation in response to adopted immune cell function against tumor cells. The phrase “adoptive immunotherapy” was originated from that case. Later, it was elucidated that T-cells accompanied by natural killer (NK) cells had the principal role in that observed phenomenon [10].

Immunotherapeutic approaches can be classified into two main categories (1) indirect modification of T-cell’s regulatory elements or immunologically active proteins like interferons, and (2) direct ex vivo manipulation and restoration of T-cells or implanting engineered universal T-cells [4]. Initial cancer immunotherapy trials have been majorly performed by using some antibodies such as ipilimumab (CTLA-4 targeting antibody), anti-programmed cell death 1 (anti-PD-1), anti-programmed death-ligand 1 (anti-PD-L1), and anti 4-1BB [11], alongside with the administration of cancer vaccines like trastuzumab emtansine for advanced her2^+^ breast cancer [12], NCS-DNA E7 vaccine against cervical cancer [13], and atezolizumab for non-small cell lung cancer [14]. Afterwards, the development of novel combinatorial methods exhibited more reliable and efficient anti-tumor responses in comparison with their separate application [15]. In this context, administration of HPV16 E7 DNA vaccine adjuvanted with anti-PD-1 and secondary lymphoid-tissue chemokine (CCL21 or SLC) or the toll like receptor agonist and α-Galactosylceramide in tumor-bearing mice models resulted in both tumor regression and tumor growth suppression [16, 17].

With the advent of synthetic biology and novel gene-therapy techniques and in the light of more efficient gene delivery tools, cancer immunotherapy has shifted into a modern era [18], where engineered autologous or adopted T-cells are implanted into the patient’s body, a cancer immunotherapy method called adoptive cell therapy (ACT). ACT is followed through three major methods including (1) using TILs, (2) modifying TCR T-cells, and (3) engineering CAR T-cells [19].

TIL contains the biopsy and extraction of T lymphocytes present in cancerous tissues, followed by culturing and activation of potentially involved T-cells. Using the autologous inactive yet potential T-cells was the initial ideahowever, the achievement to not only adequate but also appropriate and healthy tumor-specific T-cells is not always possible. Preparation of efficient T-cells by this method is time-consuming and together with intolerability against tumor changes make its application limited mostly to melanoma [20].

**Fig. 1:**
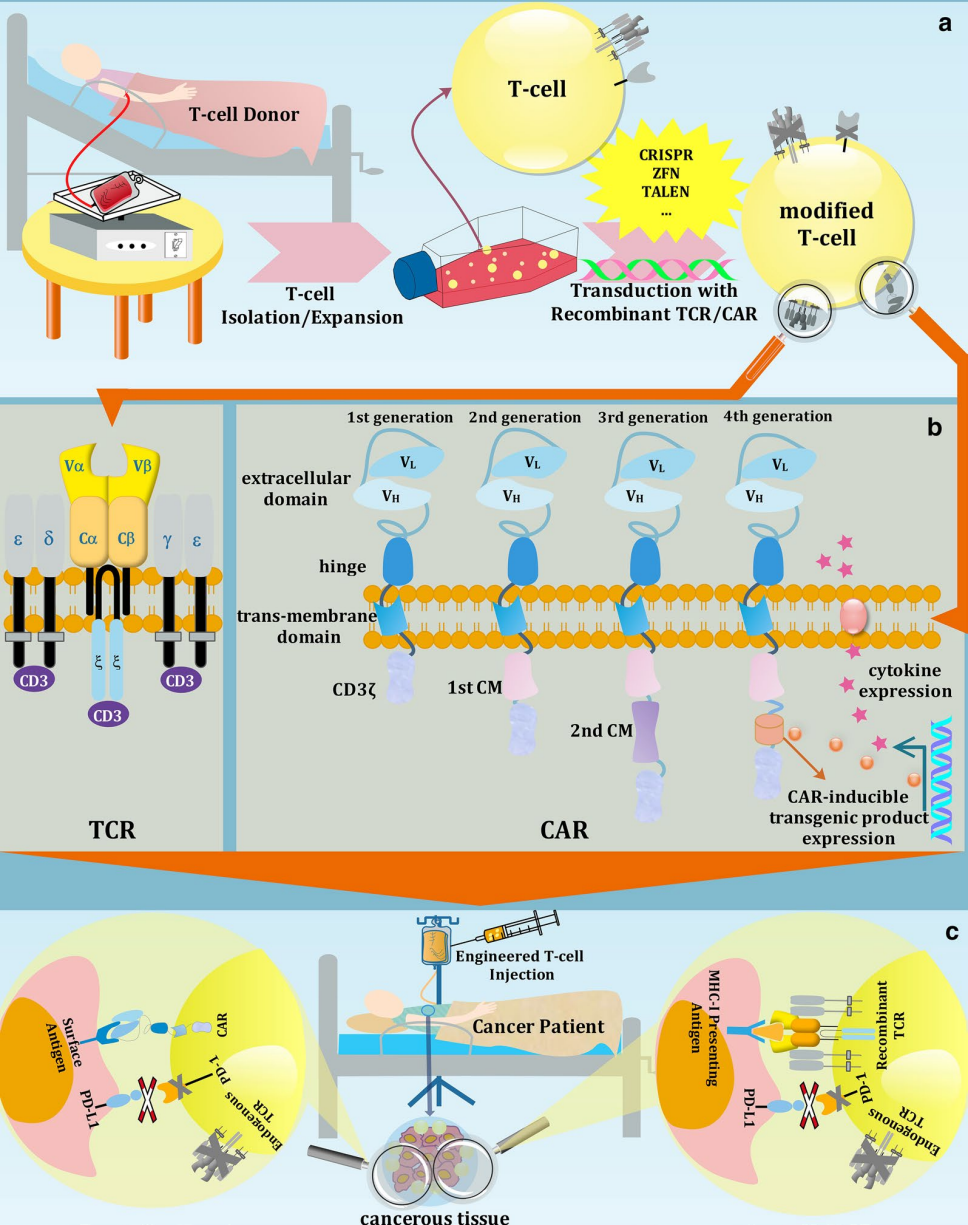
TCR and CAR T-cell cancer immunotherapy. **a** Schematic overview of CAR and TCR T-cell construction. Isolation and expansion of human T Lymphocytes (extracted from healthy donor blood) are followed by modification of T-cells by gene-editing tools (CRISPR, ZFN,…) and transduction of T-cells with transgenic TCR or CAR gene constructs. **b** TCR and four generations of CAR structure. (CM: co-stimulatory molecule, Cα: constant α-chain, Cß: constant ß-chain, Vα: variable α-chain, Vß: variable ß-chain, VL: variable light chain, VH: variable heavy chain) **c** Engineered TCR and CAR T-cells employment in the treatment of candidate patient. TCR identifies MHC-I presenting antigens and CAR targets tumor cell surface antigens

On the other hand, equipment of donor-independent T-cells with either surface antigen receptors (in CAR) or recombinant MHC-dependent antigen receptors (in TCR), is a further step to expand the application of cancer immunotherapy to cure solid tumors besides hematological malignancies (Fig. 1) [21]. Native T-cell receptors harness TCR α- and ß-chains of tumor-specific antigen as the wild type recognition element (Fig. 1b), however, the application of recombinant TCR T-cells is restricted due to their MHC-dependent tumor-independent behavior, i.e., the high probability of off-target activities [22].

Despite TCR, CAR T-cell is profiting from MHC-independent design, by which it can recognize a wide variety of cell surface antigens (protein, carbohydrate or glycolipid) [23]. Recombinant CAR is constructed by fusing extracellular single chain antibody (typically single-chain fragment antibodyscFv) with intracellular signaling domains through the hinge and transmembrane domains [24]. Evolutionary pathway of CARs consists of four subsequent generations (Fig. 1B). The first and simplest version of CARs utilized TCR-CD3ζ or FCR-ζ as intracellular signaling part. In-vivo malfunction of this generation due to lack of costimulatory signaling domains pushed the scientists to armor CARs with responding proteins such as CD28 or 4-1BB (CD137) to give birth to the 2nd generation of CARs. The 3rd generation of CARs was simultaneously equipped with two costimulatory domains [25]. Recently, the 4th generation of CARs, namely CAR-modified T-cell (TRUCK T-cell), with improved expansion and persistence to the immunosuppressive tumor environment has been developed. Potent impact of some cytokines and ligands such as IL-12 and CD40 ligand on the enhancement of T-cell activation was the initial sparkle for the genesis of this late generation of CARs [26].

In order to enhance the tumor-specific antigen recognition by CAR T-cells and to inhibit misleading of engineered CAR T-cells toward tumor-like antigen-presenting healthy cells, Kloss et al. simultaneously equipped CAR T-cells with two distinct antigen-specific receptors, one against prostate stem cell antigen (PSCA) as conventional CAR receptor and the other against prostate-specific membrane antigen (PSMA) as a chimeric costimulatory receptor, and constructed dual-targeting CAR T-cells specialized against PSCA^+^ PSMA^+^ prostate tumor cells, succeeded to efficiently reduce the risk of on-target off-tumor activity of CAR T-cell therapy in vivo [27].

The initial promising outcomes regarding CAR T-cell therapy come from targeting CD19 receptor on the surface of CD19^+^ B cell tumors [28]. The efficacy of CAR immunotherapy has been evaluated against some models of B cell cancer including malignant acute lymphoid leukemia (ALL), non-Hodgkin lymphoma, and chronic lymphocytic leukemia, and some of them have gotten FDA approval for clinical exploitation. Expansion of CAR application to the treatment of CD19^-^ hematological malignancies as well as pernicious solid tumors has been being under intensive study [29, 30].

## CRISPR, the treasure trove of gene-editing technologies

Clustered Regularly Interspaced Short Palindromic Repeats accompanied by Cas nuclease protein (CRISPR-Cas) is defined as the chief beneficiary of RNA-guided adaptive immune system of prokaryotic cells. The first CRISPR locus was initially deciphered in 1987, but its ability to provoke adaptive immunity in the presence of Cas enzyme hadn’t been determined until 2005, and since then, the revolutionary impact of CRISPR-Cas system on genetic-based manipulation strategies has begun to develop gradually [31]. Diverse types of CRISPR system have been discovered among bacteria and archae. Until now, 2 classes (based on evolved Cas proteins), 6 types and over 35 subtypes (corresponding to signature genes) have constituted the members of CRISPR big family (Table 1) [32–37].

**Table 1:** | CRISPR Classes, types, subtypes, and their original species

Reference	Species	Subtype	Type	Class
[32]	*Archaeoglobus fulgidus* AF1859, AF1870-AF1879	I-A	I	1
	*Clostridium kluyveri* CKL_2758-CKL_2751	I-B		
	*Bacillus halodurans* BH0336-BH0342	I-C		
	*Geobacter sulfurreducens* GSU0051-GSU0054, GSU0057-GSU0058	I-U		
	*Cyanothece sp. 8802* Cyan8802_0527Cyan8802_052	I-D		
	*Escherichia coli K12* ygcB-ygbF	I-E		
	*Yersinia pseudotuberculosis* YPK_1644-YPK_1649	I-F		
[34]	*Shewanella putrefaciens CN*-*32* Sputcn32_1819-Sputcn32_1823	I-F (variant)		
	*Thioalkalivibrio sp. K90mix* TK90_2699-TK90_2703	IV	IV	
	*Rhodococcus jostii RHA1* RHA1_ro10069-RHA1_ro10072	IV (variant)		
	*Staphylococcus epidermidis* SERP2463-SERP2455	III-A	III	
	*Synechocystis sp. 6803* sII7067-sII7063	III-D		
	*Methanothermobacter thermautotrophicus* MTH328-MTH323	III-C		
	*Pyrococcus furiosus* PF1131-PF1124	III-B		
	*Marinomonas mediterranea MMB_1* Marme_0668-Marme_0677	III-B (variant)		
[35]	*Legionella pneumophila str. Paris* Ipp0160-Ipp0163	II-B	II	2
	*Streptococcus thermophilus* str0657-str0660	II-A		
	*Neisseria lactamica 020*-*06* NLA_17660-NLA_17680	II-C		
[34]	*Micrarchaeum acidiphilum ARMAN*-*1* BK997_03320-BK997_03335	II-C (variant)		
[37]	uncategorized	V-F^b^	V	
	uncategorized	V-F^a^		
	*Bacillus thuringiensis HD*-*771* BTG_31928	V-U3		
	*Rothia dentocariosa M567* HMPREF0734_01291	V-U4		
	*Cyanothece sp. PCC 8801* PCC8801_4127	V-U2		
	uncategorized	V-F^c^		
	*Gordonia otitidis* GOOTI_RS19525	V-U1		
	uncategorized	V-G		
	*Oleiphilus sp.* A3715_16885-A3715_16890	V-C		
	*Bacterium CG09_39_24* BK003_02070-BK003_02075	V-D		
	*Francisella cf. novicida Fx1* FNFX1_1431-FNFX1_1428	V-A		
	*Deltaproteobacteria bacterium* A2Z89_08250-A2Z89_08265	V-E		
	*Anabaena variabilis* Ava_2196	V-U5		
	*Alicyclobacillus acidoterrestris* N007_06525-N007_06535	V-B		
	uncategorized	V-I		
	uncategorized	V-H		
[33, 34]	*Leptotrichia shahii* B031_RS0110445	VI-A	VI	
[36]	*Eubacterium siraeum* DSM_15702	VI-D1		
	*Ruminococcus sp.* N15.MGS-57	VI-D2		
[34]	*Fusobacterium prefoetens* T364_RS0105110	VI-C		
	*Prevotella buccae* HMPREF6485_RS00335-HMPREF6485_RS00340	VI-B1		
	*Bergeyella zoohelcum* HMPREF9699_02005-HMPREF9699_02006	VI-B2		

Typically, countering against foreign invasions caused by bacteriophages and plasmids by means of CRISPR machinery is pursued in three phasesadaptation, expression, and interference. During the phase one, chromosomal integration of immature fragments of foreign DNA into the spacer region of CRISPR reconstitutes a novel spacer, acting as a genetic barcode in prospective attacks by the same invader. This adaptation step is followed by the expression phase, in which the CRISPR locus is transcribed into the precursor of CRISPR RNA (crRNA), multiunit pre-crRNA, which subsequently cleaves into the mature crRNA. Next, at the interference phase, the crRNA guides Cas nuclease to pinpoint and cleave the invader DNA or in some cases summons other nucleases to get involved. This process is accomplished by coupling of the spacer at 3′ end of crRNA with its complementary sequence on the foreign invader DNA. Of note, Presence of a short motif (2–5 bp) adjacent to crRNA target on the alien DNA (protospacer), called protospacer adjacent motif (PAM), would assure the tight binding of the crRNA-Cas complex in most types of CRISPR systems [38, 39]. Interestingly, targeting invaders in type III and some subtypes of other CRISPR-Cas systems occurs through a PAM-independent procedure [40, 41]. Type III CRISPR targets ssDNA and RNA unspecifically and is completely ineffective in action with dsDNA [42]. Moreover, PAM absence restricts viral escape, which occurs in response to single-nucleotide mutation, and eliminates the need for incorporation of multiple spacers to ensure thorough immunity [43].

The most investigated type of CRISPR is type II (belonging to class 2), in which a single multidomain DNA endonuclease from *Streptococcus pyogenes*, namely Cas9, plays the pivotal role. Of note, Cas9 is solely analogous to the crRNA-Cas complex of class 1 CRISPR family [44]. In the type II CRISPR systems, cleavage of pre-crRNA into mature crRNA is achieved through the RNase III-catalyzed annealing of pre-crRNA with trans-activating crRNA (tracrRNA) encoded by type II CRISPR, leading to the formation of tracrRNA-crRNA [45]. This complex, accoutered with a 20-nt probe at its 5′ end, search for its complement sequence on the foreign DNA that possessing a PAM at its 3′ end. Afterwards, Cas binds to PAM through its PAM-interacting domain. Finally, unwinding and cleavage of target and non-target strands are handled by the HNH and RuvC nuclease domains of Cas9 [46].

Due to the straightforward architecture of type II in comparison to other types of CRISPR, its application has been widely expanded from prokaryotes’ auto-immunity to the generation of site-specific double-strand break (DSB) at any desirable targets, both in vitro and in vivo. Moreover, chimeric single guide RNA (sgRNA) has been developed as an alternative guidance instead of both crRNA and tracRNA, which makes type II CRISPR-Cas system more simple and applicable [47].

**Fig. 2:**
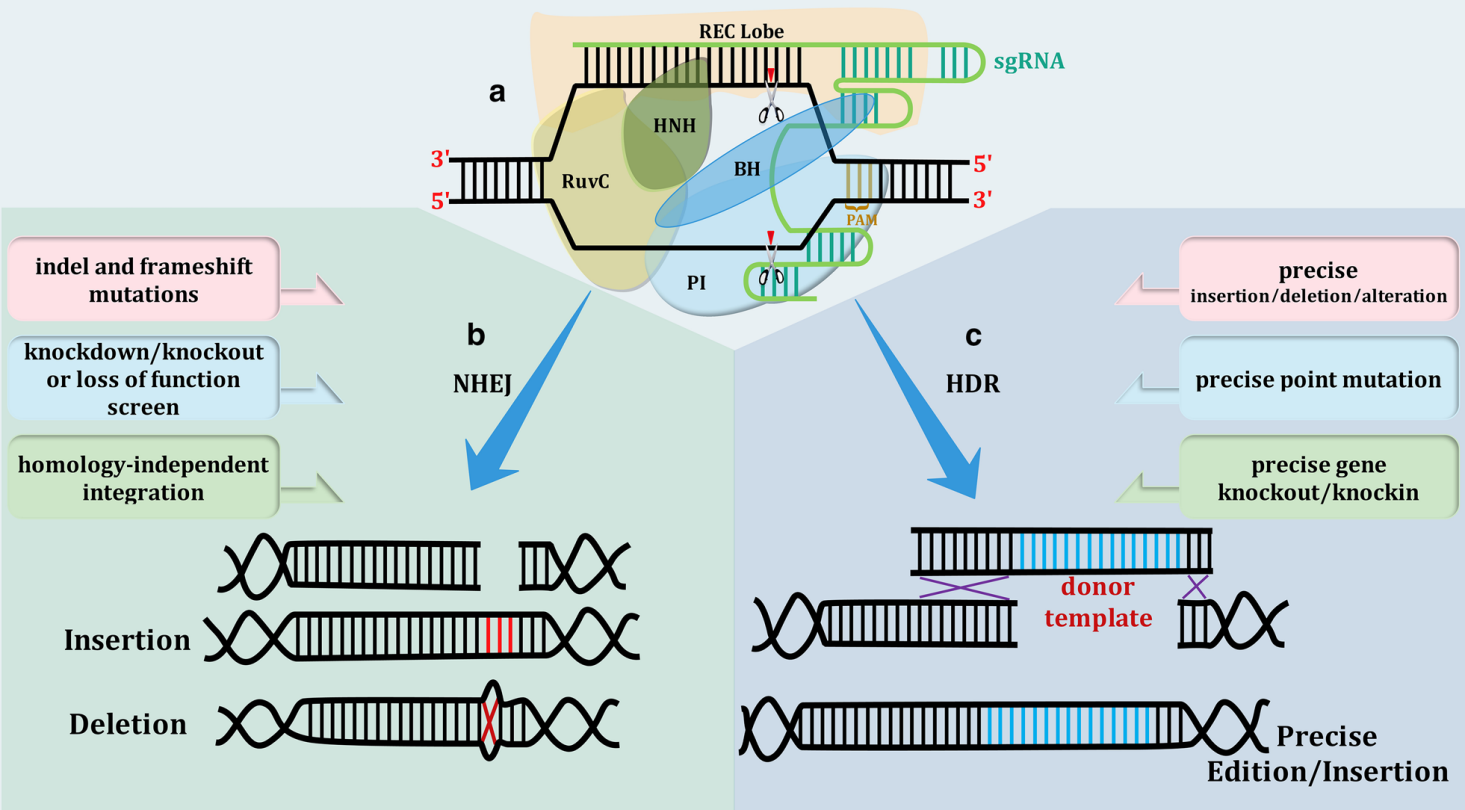
Schematic overview of the CRISPR-Cas9 complex and its mechanism of action in presence of target DNA. **a** Compartments of CRISPR-Cas9 complex bound to the target double-stranded DNA and sgRNA. Watson–Crick base pairing between target DNA and sgRNA leads Cas9 to the site of reaction. DNA DSB occurs at three nucleotides upstream of PAM motif (brown) by incorporation of HNH and RuvC domains. **b** Non-homologous end joining (NHEJ) repairing pathway and some of its consequences. NHEJ introduces indels (insertion/deletion) among target DNA and results in frameshift or loss of function inducement. **c** homology-directed repair (HDR) pathway and some of its after-effects. In presence of a donor template, which is flanked by sequences complement to target DNA, the reaction directs toward the HDR pathway, leading to precise insertion or edition

Introduction of the blunt-ended DSB is followed by activation of repairing process, the error-prone nonhomologous end joining (NHEJ) and/or the homology-directed (HDR) pathway. Depending on the presence or absence of a repairing template, HDR or NHEJ mechanism would be followed, respectively (Fig. 2). Lack of repairing template results in the activation of NHEJ pathway in which random insertions and deletions (indels), as well as substitutions at the cleavage site cause flaws in target’s function or even entirely suppress it. On the other hand, template-dependent error-free HDR route repairs the cleaved DNA target through the introduction of high precision site-specific mutation, deletion, or/and insertion by comparing the target with a template that provides the desirable modified sequence among the flanked complement nucleotides [48].

By comparison with conventional nuclease-dependent gene-editing techniques, zinc finger nuclease and transcription activator-like effector nuclease, the superiority of CRISPR-Cas nuclease method arises from its straightforward design, as well as its high efficiency and cost-effectiveness. Unlike ZFN and TALEN that their target recognition is in demand of laborious protein design and synthesis for each target, CRISPR machinery is activated by guidance of a single RNA, sgRNA. Therefore, along with noticeable reduction in the time of design and construction, RNA leadership results in feasibility of CRISPR for multi-targeting, widespread genome manipulation or screening, and adoption in different biological contexts [49, 50].

### CRISPR applications, from fundamental studies to clinical trials

CRISPR is widely used for many purposes; all are defined based on robust ability of CRISPR in gene editing. This technology not only has already found its way into molecular biology and genetic studies but also has penetrated among medical and pharmaceutical investigations [39]. Simplicity in design and feasibility in employment have paved the way for CRISPR to be exploited in diagnosis and modification of disorders at the level of nucleic acids. Moreover, CRISPR can be used at both genomic and epigenomic levels, for instance, live-cell chromatin imaging, gene expression control, nucleic acid detection, and epigenome editing [51]. In addition to host genome manipulation, CRISPR has been developed to combat viral infections and their subsequent maladies. Several pathogenic viruses, including hepatitis B virus, human papilloma virus (HPV), herpes virus, and human immunodeficiency virus type I have shown promising results in action with the CRISPR system and also some of these CRISPR-based treatments have entered into the clinical trials [52–56]. For instance, Zhu et al. introduced a safe method to target E7 in HPV-induced cervical cancer cells in vaginal context using poly ß-amino ester-based nanoparticles for delivery of CRISPR modules into xenograft tumor models, resulted in the knockout of E7 and thereby suppression of tumor progression [57]. Moreover, among all CRISPR-based therapeutic advancements, the utmost consideration has been given to the treatment of cancers. In addition, CRISPR is used to discover novel targets for further cancer therapy trials, by which anticancer drug targets and drug-resistant genes would be identified [6]. Furthermore, pinpointing the non-coding genome of cancer to complete the biological map of cancer along with the construction of organoid cancer models for in vitro investigations are the other preclinical applications of CRISPR in cancer area [58–60].

Besides, utilization of CRISPR has found many advocates among biomedical scientists. In addition to the genome and epigenome editing, manipulation of oncolytic viruses and modification of autologous tumor-specific T-cells by means of CRISPR-Cas system have currently been developed [61, 62].

Oncolytic virotherapy is another type of immunotherapy that employs oncolytic viruses for therapeutic purposes and can be used for treatment of a broad range of tumors [63]. As an example for the application of CRISPR in oncolytic virotherapy, CRISPR-Cas9 has been used to construct IL-15-expressing herpes simplex virus II, which showed potential in increasing the anti-tumor activity of T-cells and suppressing tumor growth in colon and gastric cancer models [64]. Moreover, CRISPR can be used in cancer vaccine studies to provide optimal environment for vaccine induction of enhanced immune responses against malignant cells. A very recent study has shown that CRISPR-mediated ablation of tumor cells’ CD47 highly increased the whole tumor cells vaccine-induced immunity in hematopoietic and solid tumor models [65]. Today, many CRISPR-Cas9-mediated clinical trials for treatment of cancers including relapsed or refractory leukemia and lymphoma (NCT03398967 and NCT03166878), Acute Lymphoblastic Leukemia/Lymphoma and non-Hodgkin Lymphoma (NCT03690011), relapsed or refractory B-cell malignancies (NCT04035434), relapsed or refractory multiple myeloma (NCT04244656), non-small cell lung cancer (NCT02793856), mesothelin-positive solid multiple tumors (NCT03545815 and NCT03747965), and EBV-associated malignancies (NCT03044743) are under evaluation [66].

### CRISPR-Cas challenges and possible solutions

Regardless of privileges associated with CRISPR-Cas system, some malfunctions of this technology and ethical concerns must meticulously be taken into account before entering the clinical stage [8].

Concerning the necessity for precise on-target activity of CRISPR-Cas, some studies have revealed that wild-type spCas9 is not potential to meet this requirement perfectly. to address this deficiency, development of some spCas9 mutants and its engineered homologs have led to the introduction of novel nucleases such as spCas9n, FokI-dCas9, and spCas9-HF1, which have shown improved efficacy in target specification compared to wt-sp-Cas9 [67–69]. Moreover, a recent study demonstrated that adding a hairpin sequence to 5′ end of sgRNA highly increased the accuracy of the CRISPR editing outcome [70]. Regarding prediction of the precision of CRISPR editing, Chakrabarti et al. showed that depending on the upstream sequence of PAM, the characteristics of CRISPR is predictable to some extent, allowing researchers to increase the CRISPR accuracy by following some simple rules in the design of sgRNA [71].

Another concern related to CRISPR is the ratio of HDR to NHEJ, which directly affects the efficiency of in vivo gene editing. As previously mentioned, the NHEJ repairing mechanism results in the emergence of indels among target sequence. Many trials have been conducted to enhance the portion of HDR following DSB generation. As HDR mechanism is majorly limited to S and G2 phases of cell cycle and therefore is dominated by the NHEJ pathway, many approaches including incorporation of single-stranded DNA donors, NHEJ suppressor molecules, and HDR enhancer molecules can be applied to increase the HDR ratio and thereby to improve the precision of CRISPR-Cas genome editing [72]. Moreover, postponement of initial Cas9 expression until S or G2 phases increases HDR:NHEJ ratio [73].

One of the most important problems immediately related to any gene therapy or gene drive methods is delivery. Many delivery tools have been developed, each one has its own advantageous and disadvantageous [74]. Generally, delivery methods by which CRISPR function has been assessed are categorized as physical, virally driven and non-viral vector methods. CRISPR machinery at the DNA level is delivered through viral vectors when long term activity be in demand. This method has the potential of high penetration into the target cells, and of being under tight control of an expression promoter that uses transcriptional machinery of host cells. Related to the type of virus, some points should be considered in order to prevent undesirable outcomes. Adeno-associated viruses (AAVs) are able to carry just about 4.5-5 kbp per particle, whereas the size of just spCas9 plus sgRNA is about 4.2 Kbp. Moreover, while lentiviruses and adenoviruses are benefiting from larger capacity than AAVs, the possibility of their undesired integration into the host genome as well as induction of strong immune responses are the major drawbacks of these two delivery tools [75, 76]. On the other hand, physical delivery techniques such as microinjection, electroporation, and hydrodynamic delivery are needless of any intermediate medium for delivery and activation, however, in vivo studies have disclosed their low efficiency to carry DNA particles to the target cells [77]. Among non-viral delivery methods, lipid nanoparticles have shown long-lasting stability and immune system compatibility. Other later developed non-viral delivery systems assayed at CRISPR gene-editing studies contain lipoplexes and polyplexes, cell-penetrating peptides, DNA nanoclew, induced transduction by osmocytosis and propanebetaine and gold nanoparticles [76, 78–80].

There are some other CRISPR-related concerns such as runaway immune responses and spatiotemporal malfunctions, which result in off-targeting and the decrease of productivity, which have found solutions like development of engineered or split Cas enzymes in order to have more control on in vivo activity of CRISPR-Cas system [81].

What must be considered over and above all mentioned challenges are ethical points. The tremendous need for the development of more effective and tolerable therapeutic methods to combat with ever-increasing malignancies should not lead to indiscriminate employment of those techniques before answering to all ethical questions created during initial trials, otherwise, it would immediately backfire [82, 83]. Induction of tumor suppressive protein P53 impairment, and human somatic tissues or germline cells editing, whether intentional or unintentional, are among the ethical debates aroused around CRISPR technology, which are enough to elucidate the importance of the subject [84, 85]. Additionally, some irresponsible deployment of CRISPR like the birth of CRISPR gene-manipulated twins in China should be prevented before the same fate as vaccines befalls CRISPR and stops it from its potential development [8, 86].

## The deluxe zone of cancer therapy, where CRISPR meets immunotherapy

In order to push the autologous T-cells to their limits and beyond, engineered TCR and recombinant CAR T-cells have been developed. To enhance the function and customize the properties of T-cells according to the specific tumor type, original T-cells must be modified by means of a gene-editing tool before further procedures (Fig. 1a). It is the place where CRISPR gene-editing technology overlaps with progressive cancer immunotherapy [87].

### Applications of CRISPR in construction of recombinant TCR and CAR T-cells

Embryonic T-cells, primary materials for construction of engineered T-cells, comprise native TCR complex that presents its own antigen specificity, which may interfere with both TCR and CAR T-cell therapies [88]. The potential of endogenous TCR to compete with recombinant transgenic therapeutic TCR in surface expression along with the likelihood of mixed TCR dimers formation (endogenous α with recombinant ß and vice versa) may result in poor expression of desired TCR among four possible combinations [89], and provocation of graft versus host disease (GVHD)-like syndrome [90]. Furthermore, in the context of universal T-cells, particularly CAR T-cells, the total number of expressed endogenous TCR heterodimers (either αß-TCR or γδ-TCR) on the surface of physiological T-cells may be enough to provoke GVHD [91]. To avoid these common problems, many approaches have been investigated, among which the employment of gene-editing tools (ZFN, TALEN, and CRISPR) to knock out, knock down or modify endogenous TCR is the principal technique [92, 93]. Assessment of endogenous TCR manipulation by incorporation of three distinct nuclease-based gene-editing tools by Osborn et al. revealed that the CRISPR-Cas9 complex is able to disrupt the TRAC locus of TCR-α chain efficiently without any significant off-target activity or cytotoxicity. Subsequently, these CRISPR-mediated TRAC-knockout T-cells were employed to generate efficient CD19 CAR T-cells with promising results [94]. In another study, Legut et al. showed that CRISPR-mediated knockout of the endogenous TCR-ß chain is enough to enhance the expression level of either αß or γδ recombinant TCR heterodimers. This observation can be explained by the low tendency toward formation of dimer between intact α chain of endogenous TCR with ß chain of engineered TCR in αß-TCR models and also incapability of cross-dimerization between αß and γδ chains in γδ-TCR constructs [62]. Recently, CRISPR-driven knockout of both TRAC and TRBC, to disrupt exogenous α and ß chains, respectively, for construction of transgenic TCR T-cell was evaluated. This double knockout led to the enhancement of both expression and function of recombinant TCR T-cells and hence increased their potential in antigen-sensing and control of tumor growth in multiple myeloma models compared to homologous single-locus-(TRAC or TRBC)-depleted TCR T-cells [95].

**Fig. 3:**
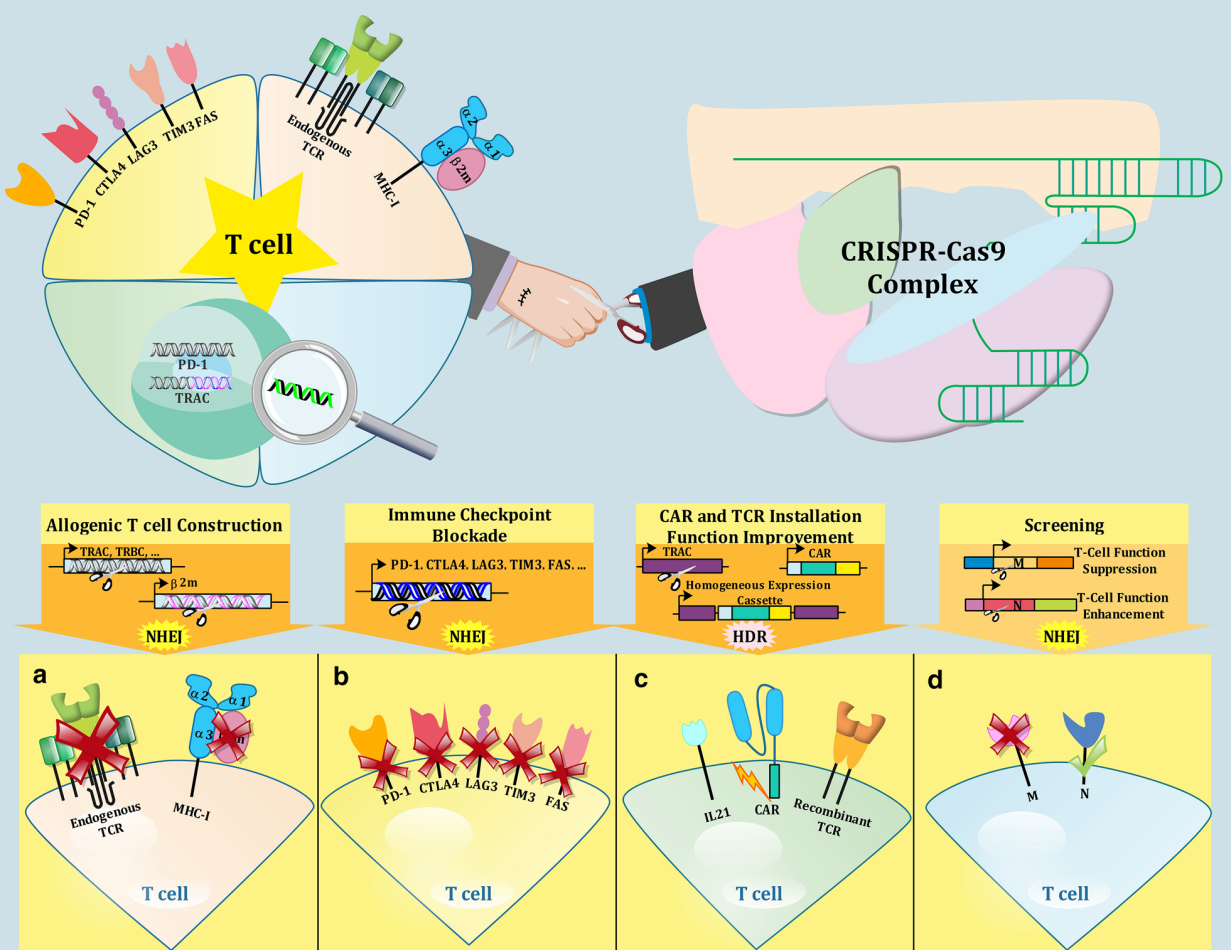
Application of CRISPR-Cas9 in construction of TCR and CAR T-cells. **a** CRISPR-Cas9 is used for the creation of allogeneic T-cells by depletion of endogenous TCR and MHC class I genes through the NHEJ pathway. **b** Inhibition of immune checkpoint pathways such as PD-1, CTLA4, LAG3, TIM3, and FAS is accessible through the employment of CRISPR-Cas9 to disrupt the related receptors on T-cells. **c** Installation of transgenic TCR or CAR into T-cells through HDR-mediated knock-in potential of CRISPR-Cas9. TCR and CAR can be inserted in between of a locus of interest. For instance, the introduction of TCR or CAR among TRAC locus in order to simultaneous knockout of endogenous TCR and establishment of recombinant TCR or CAR leads to homogenous expression of cassette and also the elimination of mutation probability, which is common among vector integration methods. **d** Loss of function screening is another application of CRISPR, in which effect of T-cell function on cancer immunotherapy in presence or absence of a gene is investigated and the results may use for development of further immunotherapeutic approaches

As CAR T-cells act particularly against tumor surface antigens and are independent of host genetics [96], they are one of the best candidates to construct universal off-the-shelf immunotherapeutic agents for rapid and ready-to-use clinical applications [97]. In addition to the previously described necessity to knock out the endogenous TCR, human leukocyte antigen (HLA) expression on the surface of universal CAR T-cells should be silenced, otherwise, the immune system would immediately reject allogeneic T-cells [98]. Furthermore, immune checkpoint blockade of inhibitory receptors (PD-1, Tim-3, CTLA-4, LAG-3, DGK, FAS, etc.) postpones CAR T-cells exhaustion and reinforces them for clinical trials [99]. Both HLA knockout and immunosuppressive pathways blockade can be achieved by means of CRISPR without any detrimental immunological side-effects as observed during employment of blocking antibodies (Fig. 3a, b) [100, 101].

Ren et al. are the pioneers of harnessing CRISPR unique ability in hitting multiple targets at once to construct allogeneic universal CAR T-cells. First, they disclosed that fueling CRISPR machinery with six distinct gRNAs through electroporation to target TRAC, TRBC, ß2 M (betta-2 Microglobulin, a subunit of HLA-I), and PD-1 results in the generation of allogenic universal CAR T-cells that are immune from stimulation of GVHD [102]. Inspired by their first innovative design, they tried adding FAS blockade gRNA to their recipe to exploit more from the CRISPR power [103]. FAS (APO-1 or CD95) receptor is expressed on extracted T-cells and its interaction with apoptosis-inducing ligand FASL, present within most human tumor microenvironments, might lead to the exhaustion of CAR T-cells and decrease in their anti-tumor function [104]. The FAS^-^ CD19 CAR T-cells, generated by simultaneous triple targeting of exogenous TCR, HLA-I, and FAS receptor in presence of eSPCas9 (the high fidelity version of Cas9) showed promising results in both in vitro and in vivo assays. However, when the number of CRISPR targets increased to include PD-1 and CTLA4 checkpoints, the yield of disruption dropped. This decline was due to the congestion of different gRNAs to compete for the use of Cas enzyme and also the limitations associated with the lentiviral vector delivery method [103]. In another study on lung cancer models, ß2 m blockade by applying CRISPR-Cas resulted in resistance to immune checkpoint inhibitors (ICIs), especially PD-1, proving the effect of HLA-I suppression on the tumor responses to ICIs [105]. Rupp et al. reported that CRISPR-Cas9-mediated Pdcd1 (PD-1) suppression in anti-CD19 CAR T-cells augments their effectiveness in destroying cancer cells in vitro using Human CD19 K562^+^ myelogenous leukemia cell line as a model, and also improves PD-L1^+^ tumor clearance in mouse xenograft models [106]. Interestingly, utilizing CRISPR to turn off the PD-L1 gene in two tumor models, MC38 and CT26, revealed that in addition to cancerous tissues, infiltrating immune cells express PD-L1 in tumor-bearing mice models [107]. The result points out the necessity of multidimensional view for designing therapeutic tools for cancer immunotherapy purposes.

Regardless of Ren et al. studies that aimed at targeting multiple sites using distinct gRNAs for each target in one-shot CRISPR treatment, Eyquem et al. introduced a novel method to create TCR^-^ CD19 CAR T-cells through single target manipulation. This study demonstrated that CRISPR-mediated insertion of the CD-19 CAR into the exon 1 of the TCR α chain (the TRAC locus) not only establishes the CAR machinery properly but also knocks out the exogenous TCR simultaneously (Fig. 3c). Moreover, using this novel construct, namely TRAC-CAR, in the treatment of precursor-B acute lymphoblastic leukemia NALM-6 mice models showed a significant decrease in expression of three important exhaustive markers (PD-1, LAG3, and TIM3) from 50% in routine CARs to below 2% in this redesigned version [108]. In order to minimize the heterogeneity among recombinant T-cells, Georgiadis et al. innovated a procedure through which Pol III- and TRAC-targeting sgRNAs were assembled with CD19 CAR into a self-inactivating lentiviral vector. Simultaneous expression of this recombinant vector with electroporation of Cas9 mRNA led to a homogenous response of Terminal-TRAC CAR T-cells against leukemia in Human xenograft tumor models [109]. Unlike the previously mentioned studies that employed electroporation for CRISPR-mediated ablation of troublesome receptors of T cells, Hu et al. evaluated nucleofection as a simpler and more robust alternative technique to disrupt PD-1 receptor and PiggyBac transposon in CD133 CAR T-cells. This method yielded more than 90 percent disruption of PD-1 and the recombinant CARs showed better anti-glioma responses both in vitro and in mice models compared to conventional CD133 CAR T-cell immunotherapy [110].

To address the concern associated with isolation and sufficient expansion of CRISPR-modified T-cells, Shao et al. [111] indicated that due to notable cytotoxic effector properties, engineered cells for costimulatory enhancement [112] in combination with Interleukin 21 provided a rich media for PD-1-disrupted (by CRISPR) cytotoxic T lymphocytes culture and thereby resulted in improvement of immune responses against AGS-EBV cell line in vitro [111].

Identification of cancer cells by CARs is limited to the recognition of membrane antigens, which contain only below one percent of all expressed proteins. This limitation create a barrier to the vast deployment of CAR T-cells in cancer immunotherapy [113]. The primary concern related to this deficiency is the probability of the presence of the same target antigen on the surface of healthy cells, which may give rise to the so called “on-target, off-tumor” phenomenon [114]. For instance, both healthy and cancerous myeloid cells express CD33 and therefore traditional CD33 CAR T-cell therapy of acute myeloid leukemia (AML), which is in phase I and I/II clinical trials, would disrupt non-cancerous normal cells too [115, 116]. Whereas tumor-specific antigen has not been identified for many tumors like some types of AML [117], and in order to prevent previously described on-target, off-tumor occurrence, two studies have demonstrated that baring the hematopoietic stem and progenitor cells from CD33 using CRISPR-Cas9 and successful transplantation of these CD33-knockout cells resulted in the safe and fully on-target, on-tumor activity of CD33-targeted CAR T-cells against AML [118, 119]. On the other hand, CD7 is a specific potential target for AML cells, but is observed in about 30% of all patients. Using CRISPR to disrupt interfering CD7 gene in CD7 CAR T-cells and to create CD7-knockout KG-1a cell line as control, Gomes-Silva et al. succeeded in the development of CD7 CAR against AML [120]. Recently, CAR T-cells targeting CD79b receptor, alone or combined with CD19, have been developed against B-cell lymphomas. And evaluation of these novel CARs in vitro and in xenograft models using CRISPR-mediated CD19^-^ MCL cell lines showed favorable results in avoiding CD19 antigen escape, reported in previous CD19 CAR T-cell therapies of B-cell lymphomas [121].

In order to broaden CAR applications from the treatment of hematological malignancies to solid tumors, utilization of appropriate antigen receptors and suppression of some limiting immune checkpoints are integral [122]. In this context, Hu et al. constructed PD-1^-^ Meso (Mesothelin) CAR T-cells using CRISPR-Cas9 to apply against TNBC, one of the most malign types of breast cancer resistant to hormonal therapy or therapies dealing with HER2 protein receptors. They observed that CRISPR-mediated PD-1-disrupted Meso CARs perform superior to untreated control Meso CARs [123]. Moreover, TIL therapy of EBV^+^ gastric cancer using CRISPR to generate PD-1^-^ T-cells opened a promising window for the treatment of chemotherapy-resistant type of gastric cancer [124]. Using the same approach, Guo et al. indicated that Hepatocellular Carcinoma cells are totally incapable of tolerating CRISPR-mediated PD-1^-^ GPC3 CAR T-cell therapy [125]. Very recently and for the first time in solid tumors, study on a recombinant anti-EGFRvIII CAR T-cell through co-disruption of TRAC, ß2 M and PDCD1 genes by means of CRISPR-Cas9 showed higher anti-glioma activities compared to previous anti-EGFRvIII CAR T-cell therapies [126]. Similarly, and this time CRISPR-Cas9-based knockout of PD-1 resulted in efficient anti-EGFRvIII CAR T-cells against glioblastoma, providing promising results for future clinical trials [127]. As previously mentioned, CAR T-cell exhaustion due to exposure to immunosuppressive microenvironment of tumor cells is an important drawback of deploying CAR T-cells against solid tumors. In this context, CRISPR-Cas9-mediated disruption of TGF-β receptor II gave rise to the production of highly efficient CAR T-cells with resistance to exhaustion, when used against solid tumors whose microenvironment is rich in TGF-β. These modified CAR T-cells also induced a decline in the TGF-β-induced conversion of regulatory T (Treg) cells [128]. In addition to CAR T-cells, using CRISPR-modified tumor-specific effector memory T-cells can be considered as an appropriate alternative for immunotherapy of solid tumors [129].

### Assisting applications of CRISPR in development of cancer immunotherapy

CRISPR plays a key role in preclinical studies of modern cancer immunotherapy. In this context, Qin et al. used CRISPR to create CD19^-^ or CD22^-^ B-acute lymphoblastic leukemia NALM6 cell line in order to control the efficiency of their double-barreled CD19 CD22 CAR. In spite of reported challenges in the establishment of both receptors, the results of that study are promising in development and optimization of novel multi-targeting CARs, which not only reduce the possibility of antigen escape but also increase the efficacy of CAR T-cell therapy [130].

Resistance to immune checkpoint blocking therapy is a prevalent phenomenon observed in many tumors, in particular, “cold” tumors, a term attributed to tumors whose microenvironment is not appropriate for infiltration of tumor-specific T-cells [131]. In this regard, Tu et al. developed a weak-acidity-responsive nanoparticle for efficient delivery of cyclin-dependent kinase 5-targeting CRISPR-Cas9 (to suppress PD-L1 expression on tumor cells [132]) and paclitaxel (to trigger anti-tumor immune responses) to the tumor site, and thereby succeeded to exchange tumor microenvironment from “cold” to “hot” and to inhibit tumor growth in melanoma and colorectal cancer mice models [133].

### CRISPR-mediated screening for potential immunotherapeutic targets

CRISPR has also been used for genome screening to determine regulatory elements that play vital roles in innate and adaptive immune pathways associated with cancer, which may be applicable for cancer immunotherapy (Fig. 3d) [134, 135]. Shifrut et al. conducted pooled screening for loss-of-function T-cells through lentiviral-based sgRNA delivery vectors and Cas9 electroporation in order to find key regulatory elements of TCR response. That study revealed that the knockout of some T-cells regulatory proteins such as SOCS1, TCEB2, RASA2 and CBLB improves both proliferation and anticancer capability of modified T-cells [136]. To explain how three subunits of PBAF (Polybromo-associated BRG1-associated factor) chromatin-remodeling complex, including ARID2, PBRM1 and BRD7 regulate T-cell effectiveness against tumor cells, Pan et al. used CRISPR to knock out each of those subunits separately in B16F10 cell line. Exposure of CRISPR gRNA library transduced B16F10^Cas9^ cells to cytotoxic T-cells indicated an enhancement in T-cell-mediated cytotoxic effect on impaired PBAF cells and in secretion of IFN-γ-inducing chemokines such as Cxcl9 and Cxcl10, leading to augmented activity of T-cells and antitumor responses [137]. In a pooled CRISPR-Cas9 mutagenesis screening, it has been demonstrated that suppression of REGNASE-1 enhanced CD8^+^ T-cells anti-tumor functions. Further analysis also determined BATF as the principal target of REGNASE-1 and introduced PTPN2 and SOCS1 as factors whose targeting by CRISPR improves the efficiency of REGNASE-1^-^ CD8^+^ T-cells [138]. CRISPR screening has also clarified the impeding role of anti-silencing function 1A histone chaperone in anti-PD-1 immunotherapy of KRAS-mutant lung adenocarcinoma patients [139].

Interestingly, CRISPR-Cas9 genome screening has introduced major histocompatibility complex, class I-related (MR1) protein as a tumor-specific target for non-conventional TCR T-cells. The attractiveness of this discovery comes from the unvaried nature of MR1, due to its mandatory involvement in some microbial metabolism pathways, as well as MR1 expression on many cancer types cells but not healthy cells, which makes it a good candidate for ACT immunotherapy. Incorporation of MR1-recognizing TCR into patients-derived T-cells resulted in efficient destruction of melanoma cells by recombinant MR1 TCR T-cells [140].

Several CRISPR-mediated investigations have performed to determine key regulatory elements contributing to cancer immunotherapy and the results have revealed the role of granulocyte–macrophage colony-stimulating factor in CD19 CAR T-cells administration [141], TNF-α autocrine level in performance of myeloid-derived suppressor cells [142], lysosome-associated membrane protein type 2a in regulation of tumor-associated macrophages [143], histone demethylase LSD1 in stimulation of anti-tumor responses [144], transcriptional co-activator with PDZ-binding motif in PD-L1 level [145], and Foxp3^+^ Treg cells in tumor advancement [146], to name a few.

In order to determine key pathways by which tumor cells restrict T-cells effector function, Kearney et al. performed a CRISPR-based genome screening and identified targets whose knockout resulted in resistance of malignant cells against T-cell attack. The findings of that study on MC38^Ova^ and B16^Ova^ cells demonstrated that elimination of some genes from three primary pathways, including antigen presentation, IFN-γ signaling, and TNF signaling leads to resistance of tumor cells to both T and NK cells both in vitro and in vivo [147]. Moreover, study on bone marrow T-cells, extracted from pediatric ALL patients, as a potential source of lymphocytes for TIL therapy, indicated the high levels of TIM3^+^ CD4^+^ T cells, which highly increase the probability of ALL relapse. Further analysis by means of CRISPR showed a direct correlation between the level of TIM-3, thereby increased level of CD200, and malfunction of T-cells against leukemia cells [148]. Intriguingly, CRISPR-mediated permanent depletion of FOXP1 in diffuse large B-cell lymphoma cell line model, an R-CHOP resistance type of non-Hodgkin lymphoma, gave rise to upregulation of MHC Class II (I-Ab) on the cell surface and therefore increased the survival rate of tumor-bearing mice models [149]. In order to elucidate the possible combinatorial role of anti-cancer or other drugs in preventing resistance to CAR T-cell immunotherapy, i.e., immunomodulatory impacts of drugs on cytotoxic properties of effector T-cells, and to explore the mechanism of CAR T-cell cytotoxicity, a detailed in vitro loss-of-function screening by means of CRISPR has conducted recently. The results revealed that cancer genetics affects the output of CAR T-cell therapy and also introduced the key role of signaling by death receptors in cytotoxic mechanism of CAR T-cells [150].

### The semi-blindfold, first human clinical trial of CRISPR-assisted cancer immunotherapy

The previously mentioned investigations were restricted to in vitro or non-human in vivo studies. First CRISPR-mediated human cancer immunotherapy was conducted in 2016 by collaboration of three groups of experts, but after very limited preclinical evaluation. In that phase I human trial, the tumor antigen NY-ESO-1 was selected to construct recombinant TCR T-cells whose endogenous TCR and PD-1 genes were knocked out by CRISPR technology [151]. These NY-ESO-1-redirected CRISPR-edited T-cells (NYCE cells) were employed for the treatment of melanoma, synovial sarcoma and multiple myeloma with the participation of six patients for each cancer type [152]. Regardless of results, many ethical issues are attributed to this semi-blindfold administration of CRISPR-manipulated T-cells [153]. Detailed research on shortages of the NYCE cell employment revealed many hidden angles of this trial. Among the results, some including maladjustment of pre-clinical and clinical conditions, restriction of pre-clinical assessment to just one case, and lack of isolation procedure to generate a homogenous cell population, characterizing PD-1^-^ TCR^KO−αß^ NY-ESO-1 T-cells, are more significant [152].

## Conclusion

Cancer immunotherapy is based on stimulation of the immune system against malignant cells in order to destroy them without any notable harmful effect on non-cancerous healthy cells. Effective function of immune system is dependent on precise identification of tumor cells by immune modules and on presence of adequate innate and adaptive immune cells in the tumor microenvironment. Initial immunotherapy trials were based on using antibodies to inhibit the immune checkpoint pathways. Regardless of some advancements in treatment of a number of cancers, some limitations such as low capacity to control the procedure of immunotherapy, inadequate native T-cell in some tumors [10, 154], and side-effects of immune checkpoint blockade [155] pushed scientists to use novel gene-editing technologies to develop customized T-cells for more efficient immunotherapy. The contemporaneousness of modern cancer immunotherapy with gene-engineering techniques has opened doors to treatment of a wide range of cancers that were considered untreatable before then. Using CRISPR to simultaneously knock out endogenous TCR, MHC-I and many immune checkpoints to construct allogeneic universal T-cells (universal TCR and CAR T-cells) have enabled scientists to focus on advanced parts of cancer immunotherapy such as the development of receptors specialized for recognition of cancer-specific antigens, as well as minimizing side-effects and off-target activity of T-cells. Many challenges such as optimal dosage, homogeneity of T-cell population to generate controlled responses, antigen escape, selection of appropriate immunotherapy target, off-target off-tumor or on-target off-tumor activity of recombinant T-cells, immune-related adverse effects, and more importantly, human-health-related ethical concerns should be addressed before accepting the engineered T-cell cancer immunotherapy as a routine clinical cancer treatment method.

## Data Availability

Not applicable.

## Abbreviations

TCR: T-cell receptor
CAR: Chimeric antigen receptor
ACT: Adoptive cell transfer
TILs: Tumor-infiltrating lymphocytes
CRISPR: Clustered regularly interspaced short palindromic repeats
ZFNs: Zinc finger nucleases
TALENs: Transcription activator-like effector nucleases
NK: Natural killer
PD-1: Programmed cell death 1
PD-L1: Programmed death-ligand 1
ALL: Acute lymphoid leukemia
crRNA: CRISPR RNA
PAM: Protospacer adjacent motif
tracrRNA: Trans-activating crRNA
DSB: Double-strand break
sgRNA: Single guide RNA
NHEJ: Nonhomologous end joining
HDR: Homology-directed
HPV: Human papilloma virus
AAVs: Adeno-associated viruses
GVHD: Graft versus host disease
HLA: Human leukocyte antigen
ß2M: Betta-2 Microglobulin
ICIs: Immune checkpoint inhibitors
AML: Acute myeloid leukemia
Treg: Regulatory T
PBAF: Polybromo-associated BRG1-associated factor
