# Correction to: CRISPR-Cas, a robust gene-editing technology in the era of modern cancer immunotherapy

**DOI:** 10.1186/s12935-020-01609-w

**Published:** 2020-10-28

**Authors:** Seyed Mohammad Miri, Elham Tafsiri, William Chi Shing Cho, Amir Ghaemi

**Affiliations:** 1 Department of Virology, Pasteur Institute of Iran, Tehran, Iran; 2 Molecular Medicine Department, Pasteur Institute of Iran, Biotechnology Research Center, Tehran, Iran; 3 Department of Clinical Oncology, Queen Elizabeth Hospital, Hong Kong, China

## Correction to: Cancer Cell Int (2020) 20:456 10.1186/s12935-020-01546-8

Following publication of the original article [1], we were notified the Department of Virology, Pasteur Institute of Iran should have been mentioned as the only affiliation for the first and last author. The revised affiliation is reflected in this correction article.
